# Where to deliver baits for deworming urban red foxes for *Echinococcus multilocularis* control: new protocol for micro-habitat modeling of fox denning requirements

**DOI:** 10.1186/1756-3305-7-357

**Published:** 2014-08-06

**Authors:** Takako Ikeda, Masashi Yoshimura, Keiichi Onoyama, Yuzaburo Oku, Nariaki Nonaka, Ken Katakura

**Affiliations:** Laboratory of Parasitology, Graduate School of Veterinary Medicine, Hokkaido University, North-18, West-9, Hokkaido, Sapporo, 060-0818 Japan; Laboratory of Wildlife Management, Obihiro University of Agriculture & Veterinary Medicine, Inada-cho, Obihiro, Hokkaido, 080-8555 Japan; Department of Entomology, California Academy of Sciences, 55 Music Concourse Drive, San Francisco, California 94118 USA; Biodiversity and Biocomplexity Unit, Okinawa Institute of Science and Technology Graduate University, 1919-1 Tancha, Onna-son, Kunigami-gun, Okinawa, 904-0495 Japan; Joint Department of Veterinary medicine, Division of Pathogenetic Veterinary Science, Faculty of Agiculture, Tottori University, Koyama, Tottori, 680-8553 Japan; Department of Veterinary Sciences, Laboratory of Veterinary Parasitic Diseases, Faculty of Agriculture, University of Miyazaki, Miyazaki, 889-2192 Japan

**Keywords:** *Echinococcus multilocularis*, Baiting strategy, Cost-benefit performance, *Vulpes vulpes*, Urban red fox, Den site selection, Key environmental factors, Key spatial scale, Requisite spatial scale, Heeding range

## Abstract

**Background:**

Deworming wild foxes by baiting with the anthelmintic praziquantel is being established as a preventive technique against environmental contamination with *Echinococcus multilocularis* eggs. Improvement of the cost-benefit performance of baiting treatment is required urgently to raise and maintain the efficacy of deworming. We established a spatial model of den site selection by urban red foxes, the definitive host, to specify the optimal micro-habitats for delivering baits in a new modeling approach modified for urban fox populations.

**Methods:**

The model was established for two cities (Obihiro and Sapporo) in Hokkaido, Japan, in which a sylvatic cycle of *E. multilocularis* is maintained. The two cities have different degrees of urbanization. The modeling process was designed to detect the best combination of key environmental factors and spatial scale that foxes pay attention to most (here named ‘heeding range’) when they select den sites. All possible models were generated using logistic regression analysis, with “presence” or “absence” of fox den as the objective variable, and nine landscape categories customized for urban environments as predictor variables to detect the best subset of predictors. This procedure was conducted for each of ten sizes of concentric circles from dens and control points to detect the best circle size. Out of all models generated, the most parsimonious model was selected using Akaike’s Information Criterion (AIC) inspection.

**Results:**

Our models suggest that fox dens in Obihiro are located at the center of a circle with 500 m radius including low percentages of wide roads, narrow roads, and occupied buildings, but high percentages of green covered areas; the dens in Sapporo within 300 m radius with low percentages of wide roads, occupied buildings, but high percentages of riverbeds and green covered areas. The variation of the models suggests the necessity of accumulating models for various types of cities in order to reveal the patterns of the model.

**Conclusions:**

Our denning models indicating suitable sites for delivering baits will improve the cost-benefit performance of the campaign. Our modeling protocol is suitable for the urban landscapes, and for extracting the heeding range when they select the den sites.

**Electronic supplementary material:**

The online version of this article (doi:10.1186/1756-3305-7-357) contains supplementary material, which is available to authorized users.

## Background

The establishment of effective strategies for zoonoses control is needed urgently in order to minimize infection risks to humans, because wildlife and human habitats are becoming rapidly overlapped [1].

*Echinococcus multilocularis* Leuckart, 1863 is a parasite perpetuated in a sylvatic cycle mainly between wild carnivores (definitive hosts) and rodents (intermediate hosts). Infection of humans occurs by the accidental ingestion of the parasite eggs, which are provided from the feces of the definitive hosts. This ingestion will cause human alveolar echinococcosis (HAE), which constitutes a serious zoonosis. The number of cases of HAE has been increasing in recent years in central Europe, parts of North America, and parts of Asia including Japan [2, 3].

In Japan, HAE is endemic in Hokkaido, the northernmost prefecture. Here, the red fox, *Vulpes vulpes* Linnaeus, 1758, is the main definitive host [4] and acts as a vector of *E. multilocularis* toward humans.

The red fox is common wildlife in Hokkaido, and it is known to have a high capacity for adaptation to artificial environments. In fact, their habitat has expanded into urban areas of many cities worldwide in recent decades [5–8]. This urbanization of red foxes has been reported in Hokkaido as well [9–12]. Moreover, *E. multilocularis* is prevalent among the urban fox population there [9, 12]. The urbanization of infected red foxes leads to contamination of these areas with eggs of *E. multilocularis* and raises the exposure risk of residents to the pathogenic eggs.

Deworming of foxes by baiting with anthelmintic praziquantel could prevent the contamination of areas with the eggs of *E. multilocularis*. Previous studies have demonstrated that this approach successfully reduced *E. multilocularis* prevalence in the red fox population in several countries [7, 13–22].

Although effective, anthelmintic baiting requires continuous effort to keep the fox population in the target area free from parasites. Even if family members are effectively treated, the risk of re-infection increases again during the annual immigration. Achieving the maximum effect at the minimum cost is fundamental for sustainable baiting, hence identifying the most suitable locations for delivering baits is necessary [23–26], especially areas having low densities of foxes such as cities in Hokkaido (e.g. 0.080 families/km^2^ in Sapporo [11]).

Clarifying the pattern of habitat use to standardize the target locations for delivering baits could improve the cost-benefit performance of anthelmintic baiting. The target location should be related to the habitat use of red foxes [8, 27, 28], especially around dens, which are the pivot of their habitation. A red fox family usually has several dens in different places and they depend on the sites throughout the breeding season. They are likely to intake bait around the dens constantly because they invariably come back at least once a day to any one of their dens during the breeding season. Indeed, a camera trap study revealed that foxes accepted baits frequently at their dens during the season [29]. Hence, the requirements for fox denning are the key to determining the target location where bait should be delivered. Standardized denning requirements could be clarified by establishing a model that extracts key environmental factors.

A general modeling method exists for standardizing the habitat selection of arthropods [25, 30–35]; however, this method is not applicable to modeling the habitat use of urban foxes. This general modeling method is appropriate for risk prediction of vector-borne diseases mechanically transmitted by arthropods, which targets the macro-scale area. On the other hand, the fox model is intended for the risk prediction of echinococcosis, which is a parasitic zoonosis indirectly transmitted by a mid-sized, generalist species inhabiting urban landscapes. Three major problems must be solved to apply the existing method to habitat use modeling for red foxes: 1) the general modeling approach uses the “abundance” of vector individuals as its modeling target; however, “presence or absence” is suitable for fox modeling especially in the areas in which they inhabit in low densities; 2) the general modeling approach uses variables on existing thematic maps, but these variables for foxes should be based on their individual habitat use, not general land use nor general vegetation; 3) the size of unit in the general modeling approach is based on the grid size (resolution) of existing thematic maps; however, neither fox territories nor their habitat use can be represented by the resolution of these maps.

In the present study, we specified the potential habitat of urban fox dens by establishing an innovative fox denning model, which identifies the suitable locations for delivering anthelmintic baits. The fox denning model was designed as a den site selection model that can extract the best combination of key environmental factors and key spatial scale for denning simultaneously. The presence or absence of fox dens was set as the modeling target, which is applicable for analyzing fox denning instead of abundance of individuals. The new modeling method simultaneously extracts the best combination of critical factors and an optimal size of modeling unit from all combinations. This is the first approach to establish a comprehensive micro-habitat model for mid-sized and generalist mammals in consideration of specifying the requisite spatial scales for the target populations. The protocol for the modeling process is presented visually. In addition to spatial modeling, a comparison is made between the results of denning factors extracted by our new model and by two traditional univariate analyses. The extracted factors by the traditional analyses are also compared with the results from other places reported in previous studies to discuss the differences in fox denning requirements depending on habitat types. Control strategies for *E. multilocularis* are also discussed.

## Methods

We established a new spatial model to specify the potential habitat of urban red fox dens to identify the suitable location to deliver anthelmintic baits. The model clarifies the critical environmental requirements for den site selection by urban red foxes. Models were constructed for urban areas of Obihiro and Sapporo cities in Hokkaido, Japan, in which red fox populations have been established. The modeling protocol is given below (see Analysis: “Den site selection modeling”).

In addition to establishment of the new model above, we extracted denning factors using two other traditional univariate analyses to compare the results between the methods. The factors extracted by the traditional approaches are also compared with the results from previous studies conducted in non-urban areas [36–43] to discuss the differences in fox denning requirements depending on habitat type. The protocols of the two traditional analyses are also given below (see Analysis: “Supplemental analyses by traditional methods”).

### Study areas

The study areas were urban regions of Obihiro and Sapporo cities in Hokkaido, the northernmost prefecture of Japan (N 41°21′-45°33′, E 139°20′-148°53′). Hokkaido belongs to the subarctic zone and shows a continental climate, and it usually snows from November to March although the annual amount of snowfall varies depending on the province. Obihiro City is a small city located in the eastern part of the island. Sapporo City is the prefectural capital and located in the western part of Hokkaido Island, and in which *E. multilocularis* has been fixed in red foxes. The densities of red foxes in urban areas of Hokkaido is relatively lower (e.g. 0.080 families/km^2^ in Sapporo [11]) than in other cities in Europe [20, 44–46]. Both of the study areas are composed almost entirely of artificial environments, including urban parks and farmland; however, these two study areas are different in the scale of each component, i.e. surface area, human population size, and human population density.

**Figure 1:**
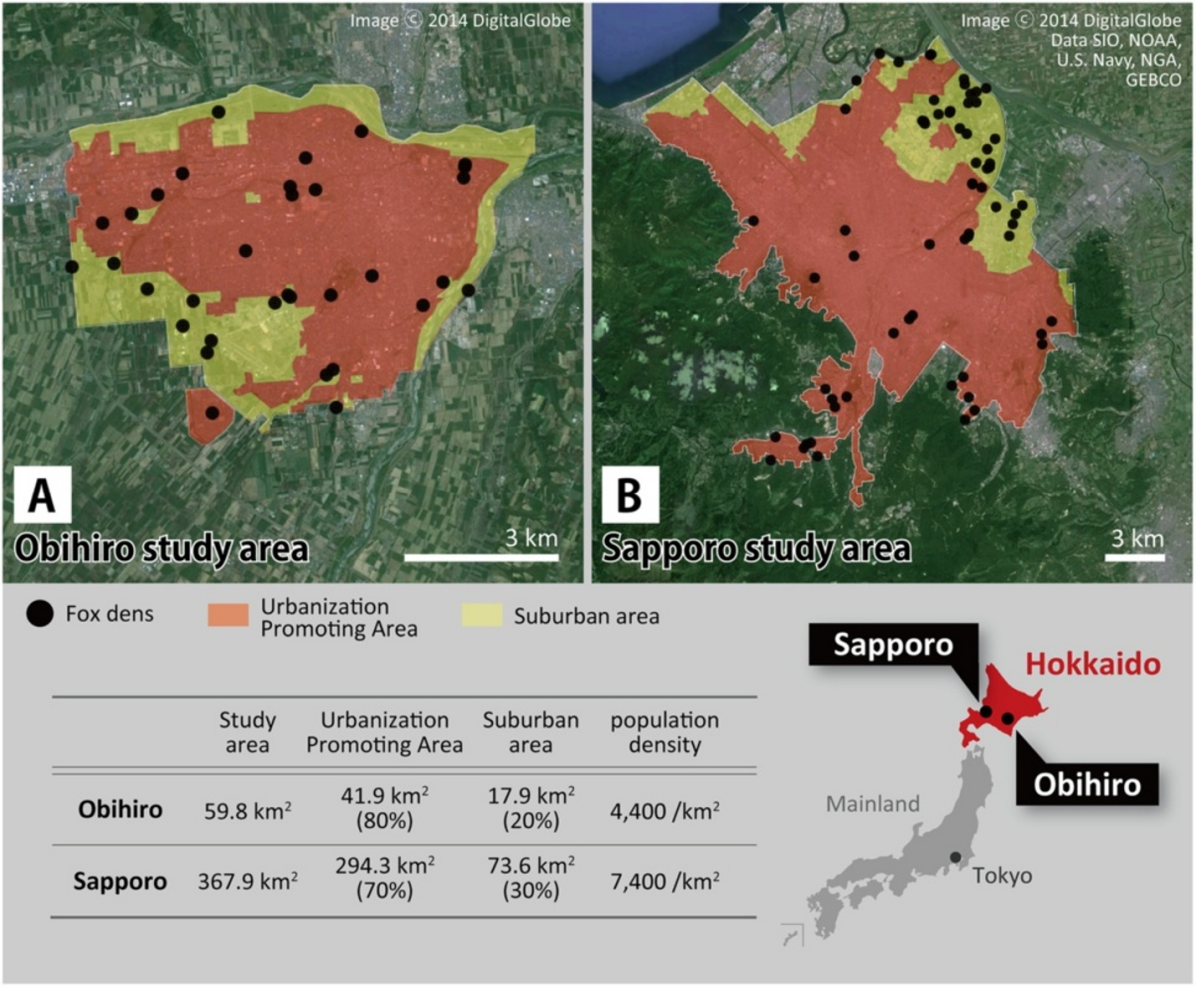
**Maps showing the landscape structures and fox den distributions in the two study areas.** Panel **A** shows Obihiro study area and panel **B** shows Sapporo study area. Each study area consists of Urbanization Promoting Area (orange area) and surrounding suburban area (yellow area). Black dots in the maps indicate red fox dens.

A map of the Obihiro study area is given in Figure 1-A. This study area (about 59.8 km^2^) consists of the whole of the Urbanization Promoting Area (UPA; about 41.9 km^2^) and its surrounding suburban area (about 17.9 km^2^). The UPA is composed of a mosaic of dwellings, commercial areas, urban parks, urban green spaces, and riverbeds. The surrounding suburban area is composed of urban parks, an area of continuous farmlands, and riverbeds of two large rivers, plus some small rivers and streams. The human population of the study area is approximately 167,000, which amounts to 96% of the total population of whole city. The population density is about 4400 people/km^2^.

A map of the Sapporo study area is given in Figure 1-B. This study area (about 367.9 km^2^) consists of the whole of the UPA (about 249.3 km^2^) and its surrounding suburban area (about 73.6 km^2^). The UPA is composed of a mosaic of dwellings, commercial areas, urban parks, urban green spaces, and riverbeds. The surrounding suburban area is composed of large urban parks, urban farmland, and riverbeds of two big rivers, plus some small rivers and streams. The human population of the study area is approximately 1,855,000, which is around 99% of the total population of the whole city. The population density is about 7400 people/km^2^.

### Analyses

#### Den site selection modeling

**Figure 2:**
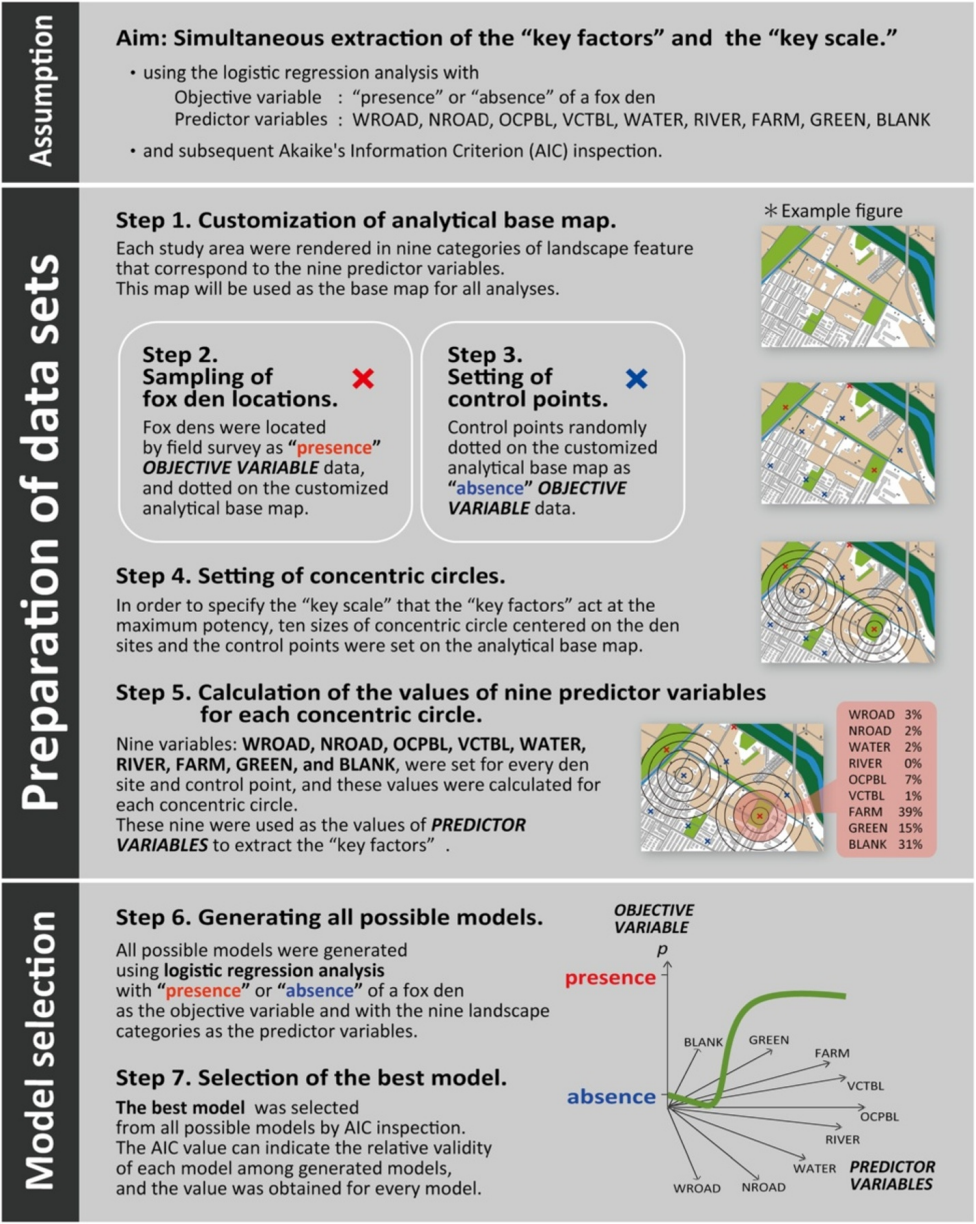
**The protocol for the modeling process.** *See also the legend of Figure 3-A.

The modeling process was designed to extract the critical environmental requirements for den site selection by urban red foxes. The environmental requirements in this study is described as the combination of the landscape factors most affecting den site selection (hereinafter referred to as “key factors”) and the most affecting spatial scale (“key scale”). The “key scale” is not the same as the home range or territory but the “heeding range”, in which they would be more nervous about disturbance and secure resources compared with outside the range within their home range. We aimed to extract the best combination of the “key factors” and the “key scale” through the modeling, which is performed by all possible subset model selection using logistic regression analysis and subsequent Akaike’s Information Criterion (AIC) inspection. The protocol for the modeling process is given below and in Figure 2.

##### Assumptions of the modeling

The regression analysis consisted of the presence or absence of a fox den as the objective variable, and nine categories of landscape features as the predictor variables. The nine predictor variables were presented by percentages of area occupied by nine categories of landscape feature: “wide road” (WROAD), “narrow road” (NROAD), “occupied building” (OCPBL), “vacant building” (VCTBL), “water place” (WATER), “riverbed” (RIVER), “farmland” (FARM), “green covered area” (GREEN), and “blank space” (BLANK). These were equipped for analyzing urban habitat use by red foxes based on previous studies on fox habitat selection [36–43]. These variables were carefully chosen to reflect the sensitivity of foxes against artificial structures when they select the den sites.

Detailed definitions of landscape feature categories and those of corresponding variables and abbreviations are shown in Table 1.

**Table 1:** **Definitions of the landscape feature categories and terms, and their corresponding variables and abbreviations**

Category of landscape feature	Definition of term	Abbreviation of modeling variable	Abbreviation of linear distance variable
Wide road*	Paved roads (≥5.5 m width) and railways.	WROAD	L-wroad
Narrow road*	Paved roads (<5.5 m width) and unpaved roads.	NROAD	L-nroad
Water place*	Rivers, streams, and drains.	WATER	L-water
Riverbed*	Vegetated or dried areas along rivers.	RIVER	L-river
Occupied building**	Buildings that are always occupied by human activity, i.e. dwelling houses, outlets, and schoolhouses.	OCPBL	L-ocpbl
Vacant building**	Buildings that are not always occupied by human activity, i.e. barns, garden sheds, and garages.	VCTBL	L-vctbl
Farmland***	Meadowlands and croplands.	FARM	L-farm
Green covered area***	Green covered areas except for riverbeds and farmlands, i.e. urban parks and urban green spaces.	GREEN	L-green
Blank space***	Remaining areas that do not have any roads, rivers, water, buildings, or vegetation.	BLANK	L-blank

*Based on definition of numerical information maps.

**Based on definition of numerical information maps and house maps.

***Extracted from aerial photographs.

##### Modeling process

The detailed modeling process is described below. The process consists of the preparation of data sets (Step 1–5) and model selection (Step 6–7). A series of modeling processes was performed using statistical software R 3.0.3 (The R Project for Statistical Computing) [47] and the R packages of all.logistic [48] and glm2 [49].

### Step 1. Customization of analytical base maps

A specialized analytical base map was prepared for each study area by customizing existing thematic maps to render the whole study area in nine categories of landscape feature: “wide road”, “narrow road”, “occupied building”, “vacant building”, “water place”, “riverbed”, “farmland”, “green covered area”, and “blank space”.

The categories “occupied building” and “vacant building” were distinguished to investigate whether foxes were sensitive to the presence of humans or artificial structures. “Water place” and “riverbed” were distinguished for detailed investigation of the reason why foxes prefer den sites near a river. It was reported previously that red foxes prefer sites near a river; however, it has not yet been clarified whether they are attracted to rivers just as a source of water or whether they are attracted to other environmental factors associated with the river, such as a riverbed with a slope and dry sand that may enable them to dig easily, fewer invaders, many rodents as food, etc. [41, 50]. The category “farmland” was distinguished from “green covered area” to determine if foxes are sensitive to disturbance by farmers or tractors.

The landscape data was referenced from several numerical information maps from the National Land Numerical Information download service (Geographical Survey Institute, Japan [51]) and the Fundamental Geospatial Data 25000 Web Map Service (Geographical Survey Institute, Japan [52]), Residential Maps (Hokkaido-Chizu Co., Ltd. [53, 54], ZENRIN Co., Ltd. [55] and ZENRIN PRINTEX Co., Ltd. [56]), aerial photographs (PHOTEC Co., Ltd. [57] and Google Earth [58]), and field inspection. The rendering process was performed using geographic information system software (free software: Quantum GIS 1.8.0, QGIS Development Team [59]), a photo-retouching software (free software: Paint.NET 3.5.10 [60]), and image analysis software (free software: Image J, U.S. National Institutes of Health [61]). The latter two were used to extract and ascertain borders of farmlands, green covered areas, and blank spaces from aerial photographs, because these landscape features were not distinguished fully in the numerical information maps.

**Figure 3:**
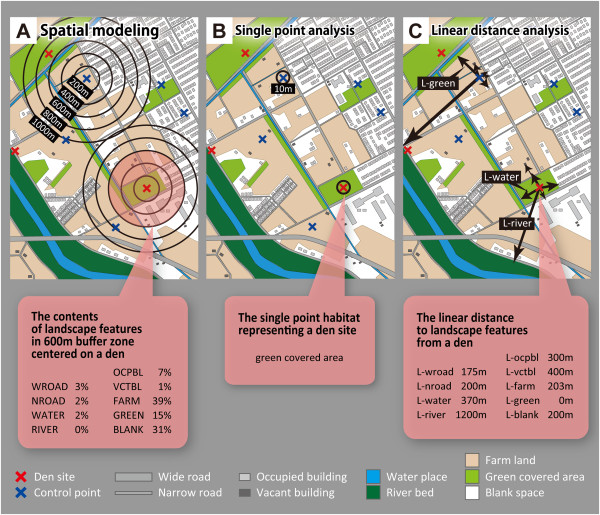
**Example of customized analytical base map and calculation methods of three types of predictor variables.** Panel **A** shows an example of the calculation of the values of nine variables for spatial modeling. Black open circles indicate ten sizes of concentric circle (100–1000 m in radius, at 100 m intervals) centered on a den site or a control point. In this figure, only circles of 200, 400, 600, 800, and 1000 m are shown. The pink circle indicates a 600 m concentric circle. Percentages of the dimensions of nine landscape feature categories included in the circle centered on a den site were calculated as shown in the pink call-out. Panel **B** shows an example of habitat determination by single point analysis. Black open circles indicate a 10 m radius circle centered on a den point or a control point. Just one habitat of a den site included in the radius is determined as shown in the pink call-out. Panel **C** shows an example of measurements of the values of nine variables for linear distance analysis. Black arrows indicate the shortest distances to the nine landscape feature categories from a den site or a control point. The distances to the nearest nine landscape features from a den site were measured as shown in the pink call-out.

These customized maps were used as the base maps for all analyses described below. Detailed definitions of the nine landscape feature categories are shown in Table 1. An example customized map is shown in Figure 3.

### Step 2. Sampling of fox den locations

Fox dens were located as “presence” values of objective variable, and den locations were dotted on the customized analytical base map.

Dens were found by exploring all vegetated areas and unpaved ground along the riverbed from 2002 to 2004 in the Obihiro study area (Figure 1-A), on the basis of the results of questionnaire surveys conducted with staff of city cleaning departments, students of twelve public junior high schools, and farming families. Exploration was carried out from 2004 to 2007 in the Sapporo study area (Figure 1-B) with the support of hunters in addition to location data collected from farmers and previous reports [9, 12]. All tunnels with a diameter of circa 20 cm excavated by animals were regarded as red fox dens [41, 62]. Another animal that may use such dens around the study areas is *Nyctereutes procyonoides* Gray, 1834 (Raccoon dog), but this is a nonnative species and has not taken root yet in the present study areas. The location data of all dens found through the field surveys were recorded using a GPS receiver (Garmin Ltd., GPS 12CX), and plotted on the customized analytical base map.

### Step 3. Setting of control points

As against the points with dens present, control points were dotted randomly on the customized analytical base map as “absence” objective variable data.

In total, 120 points in the Obihiro and 730 points in the Sapporo study areas were generated randomly as points with dens absent on the customized analytical base map. The random points were eliminated and generated newly if they were located on roads, in occupied buildings, or in water. Points on farmland were accepted as control points in this study.

### Step 4. Setting of concentric circles

In order to specify the “key scale”, ten sizes of concentric circle were set.An example of these concentric circles is shown in Figure 3-A. The circles were 100 m in radius centered on every den and control point on the analytical base map, and each circle was expanded to 200, 300, 400, 500, 600, 700, 800, 900, and 1000 m from each point in a concentric pattern (200, 400, 600, 800, 1000 m circles are shown in Figure 3-A). The “key scale” was determined from these circles. The values of variables defined in Step 5 were calculated for each circle around all den sites and control points.

### Step 5. Calculation of the values of nine variables for each concentric circle

The values of nine variables within each concentric circle set in Step 4 were calculated as predictor variables. This calculation was carried out for each size of concentric circle for every den site and control point (as in Figure 3-A: pink balloon).

### Step 6. Generating all possible models

All possible models (_9_C_1_ + _9_C_2_ + _9_C_3_ + … + _9_C_9_ = 511 models) were generated using logistic regression analyses with “presence” or “absence” of a fox den as the objective variable (see Steps 2 and 3), and nine landscape variables: WROAD, NROAD, OCPBL, VCTBL, WATER, RIVER, FARM, GREEN, and BLANK as the predictor variables (see Step 5). This procedure was conducted for each of the concentric circles (see Step 4).

### Step 7. Selection of the best model

Out of all models generated in Step 6, the most parsimonious model was selected using AIC inspection. The AIC can indicate the relative validity of each model among all the models generated. The lower the AIC value, the higher the relative validity of the model. The rank of the models can be determined using the AIC value only among the values generated for the same objective variable from the same set of predictor variables. For example, the AIC values for the models of Obihiro and Sapporo study areas cannot be compared.

#### Model validation

The best models established here were validated by the area under the curve (AUC) of the receiver operating characteristic (ROC) curve. The AUC can be used to validate the model’s accuracy [63]. AUC values range between 0.5 (low accuracy) and 1 (high accuracy).

### Supplemental analyses by traditional methods

The traditional methods (univariate analyses, not regression modeling) target only the “key factors” extraction, not the “key scale”. The nine landscape feature categories (defined in Step 1 and Table 1) and the control points (generated in Step 3) were shared with the spatial modeling protocol. The validity of these analytic methods is also discussed (see Discussion: *“*Unsuitability of traditional methods for extraction of factors in urban landscapes”).

#### Single point analysis

The first traditional analysis method regards the den site as just a “single point” habitat, not a complex of environmental features. This is the most primitive method of quantitative analysis of den site distribution pattern. This method is not capable of extracting detailed “key factors” of den site selection but is convenient for providing a brief overview of the tendencies of den site distribution. The habitat of a den site was determined using only one major landscape feature: “wide road”, “narrow road”, “occupied building”, “vacant building”, “water place”, “riverbed”, “farmland”, “green covered area”, and “blank space” in a 10 m radius centered on the den point. An example of this determination of habitat is shown in Figure 3-B. The habitat of a control point (120 points in Obihiro and 730 points in Sapporo) was determined in the same way. The habitats of den sites and habitat availability were compared by 9 × 2 G-test of fitness [64].

#### Linear distance analysis

The second traditional analysis method evaluates the disturbing or attracting factors as “linear distance” from the den site. This popular method of quantitative analysis of relative usage of landscape can be used to extract “key factors” for den site selection. Nine variables for this analysis were set. The variables representing each den point were determined as the distances from each den to the nearest “wide road” (L-wroad), “narrow road” (L-nroad), “occupied building” (L-ocpbl), “vacant building (L-vctbl)”, “water place” (L-water), “riverbed” (L-river), “farmland” (L-farm), “green covered area” (L-green), and “blank space” (L-blank). An example of the measuring method of the values of each variable is shown in Figure 3-C. Detailed definitions of landscape feature categories and the corresponding variables and abbreviations are shown in Table 1. Values of the nine variables were calculated for den sites and control points (120 points in Obihiro and 730 points in Sapporo) and the values were compared by Mann–Whitney *U* test [41].

## Results

In the Obihiro study area, a total of 35 fox dens were found (0.59 dens/2,793 people/km^2^). All dens found were tunnels excavated in the ground. Most were dug in flat ground and a few dens were on a slope.

In the Sapporo study area, a total of 65 fox dens were found (0.18 dens/5,042 people/km^2^). All dens reported in previous studies (21 dens) in 1997, 1998 [9] and 2003 [12] still existed exactly at the same location or in the close vicinity. The owners of these dens were considered to be the offspring of the previous owners, because red foxes tend to inherit the dens in which they were born and raised. The remaining 44 dens were newly found in the present study. Most dens were excavated in the ground, but eight found in the UPA were converted from artificial structures such as abandoned barns or stacks of scrap wood and building materials. For the dens dug in the ground, dens in riverbeds were on a slope but most were dug in flat ground.

### Fox den site selection model

**Figure 4:**
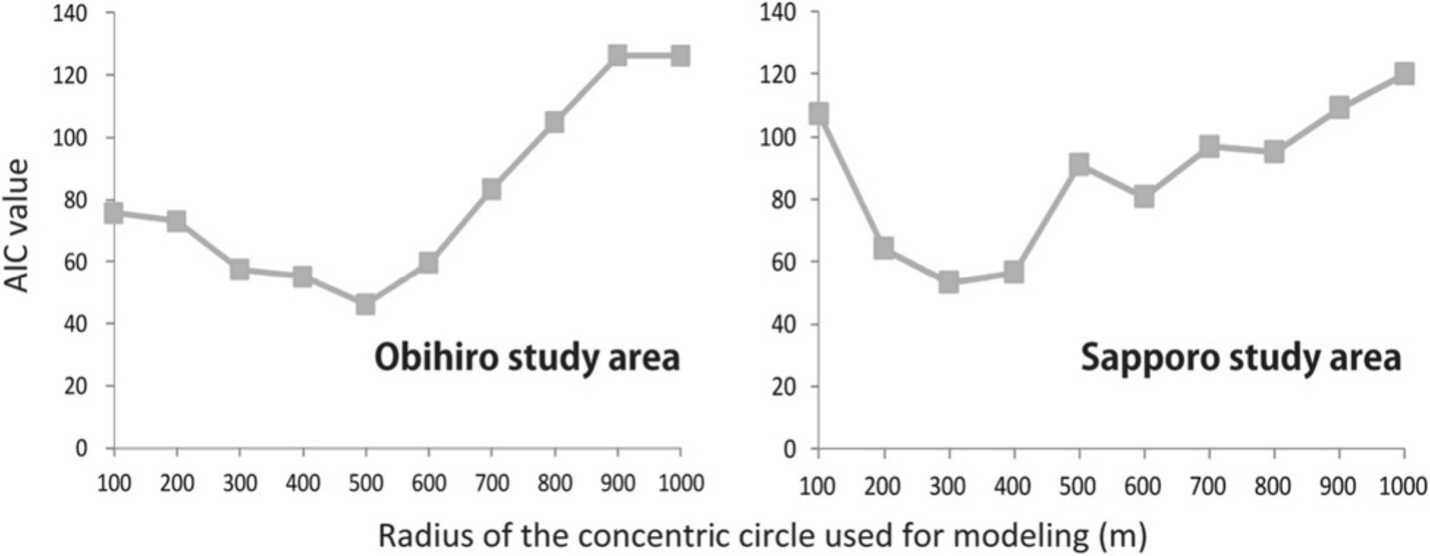
**Changes in AIC depending on the size of concentric circles in the two study areas.** Akaike’s Information Criterion (AIC) values of the best models of each of the ten sizes of concentric circles are shown. The AIC value shows a minimum at 500 m in Obihiro (left) and at 300 m in Sapporo (right).

The best model consisting of the best combination of the “key factors” and “key scale” for den site selection by foxes was determined for each city. Higher ranked models for each study area are listed in Tables 2 and 3, and changes in AIC values of the models depending on the sizes of concentric circles are shown in Figure 4. AUC values that indicate the model validity (model’s accuracy) are shown in Table 4.

**Table 2:** **Selected variables in the best models for each concentric circle in Obihiro**

Rank of model	Radius of concentric circle (m)	AIC value	ΔAIC	Intercept	Variable								
					WROAD	NROAD	WATER	RIVER	OCPBL	VCTBL	FARM	GREEN	BLANK
1	500	46.2	0.00	1.749	−0.306	−0.709	-	-	−0.338	-	-	0.613	-
2	400	55.1	8.92	−0.005	−0.180	−0.489	-	-	−0.271	-	-	0.522	-
3	300	57.5	2.39	9.205	−0.133	−0.597	-	-	−0.407	−0.184	-	0.216	-
4	600	59.7	2.14	1.808	−0.186	−0.514	-	-	−0.201	-	-	0.275	-
5	200	73.1	13.41	5.616	−0.226	−0.616	-	-	−0.280	−0.190	-	0.047	-
6	100	75.7	2.63	3.456	−0.245	−0.290	-	-	−0.222	−0.131	-	0.766	−0.162
7	700	83.4	7.67	3.466	−0.192	−0.563	-	-	−0.159	-	-	0.041	-
8	800	105.0	21.62	1.501	−0.118	−0.302	-	-	−0.147	-	-	0.145	-
9	1000	126.2	21.24	−0.222	−0.160	−0.132	-	0.438	−0.054	-	-	0.203	−0.067
10	900	126.4	0.12	2.738	−0.196	−0.196	-	-	−0.049	-	-	0.141	−0.059

The numbers under the variables are partial regression coefficients of selected variables in each model. The partial regression coefficient indicates the contribution ratio of each variable. Ranks of models are given in order of AIC values. ΔAIC in this table indicates the difference in AIC value from the higher ranked model right above.

**Table 3:** **Selected variables in the best models for each concentric circle in Sapporo**

Rank of model	Radius of concentric circle (m)	AIC value	ΔAIC	Intercept	Variable								
					WROAD	NROAD	WATER	RIVER	OCPBL	VCTBL	FARM	GREEN	BLANK
1	300	53.3	0.00	−10.167	−0.169	-	-	0.328	−0.128	-	-	0.575	-
2	400	56.5	3.25	−5.822	−0.266	-	-	0.192	−0.273	−1.022	-	0.590	-
3	200	64.2	7.69	−9.526	−0.186	-	-	0.344	−0.134	-	-	0.562	-
4	600	80.8	16.56	−8.018	−0.474	-	-	0.338	−0.235	-	-	0.422	-
5	500	91.1	10.27	−4.343	−0.314	−0.602	-	0.115	−0.093	-	-	0.476	−0.061
6	800	95.1	4.08	−4.326	−0.263	−0.353	-	0.147	−0.238	-	-	0.380	-
7	700	96.8	1.62	−2.783	−0.330	-	-	0.144	−0.300	−1.016	-	0.413	-
8	100	107.3	10.52	−2.265	−0.288	−0.724	-	0.141	−0.247	-	-	0.393	-
9	900	109.2	1.98	−8.850	−0.047	-	-	0.147	−0.259	-	-	0.645	−0.407
10	1000	120.0	10.74	−2.409	−0.268	−0.846	-	0.127	−0.270	-	-	0.419	-

The numbers under the variables are partial regression coefficients of selected variables in each model. The partial regression coefficient indicates the contribution ratio of each variable. Ranks of models are given in order of AIC values. ΔAIC in this table indicates the difference in AIC value from the higher ranked model right above.

**Table 4:** **Comparison of the models’ discriminating abilities among the variable types**

Type of modeling variable	Obihiro			Sapporo		
	Selected variables	Model AUC	Model R ^2^	Selected variables	Model AUC	Model R ^2^
Percentages of landscape features* (best model)	WROAD	0.987	0.781	WROAD	0.995	0.904
	NROAD			RIVER		
	OCPBL			OCPBL		
	GREEN			GREEN		
				L-river		
	L-river			L-ocpbl		
Linear distance	L-green	0.722	0.128	L-vctbl	0.881	0.298
				L-farm		
				L-blank		
Single point habitat	n/a**	-	-	n/a**	-	-

*The values are shown for the best model of each study area.

**Single point habitat is not applicable as the predictor variable for modeling because this variable is univariate.

In the Obihiro study area, higher ranked models produced comparatively stable variables, as shown in Table 2. The lowest AIC value was given for the model with a 500 m radius concentric circle size (Figure 4) and the accuracy of this best model is sufficiently high (AUC = 0.987; Table 4). Extracted variables within the best size of concentric circle were WROAD, NROAD, OCPBL, and GREEN. The directions of effect of these variables were minus for WROAD, NROAD, and OCPBL, and plus for GREEN depending on partial regression coefficients. The prediction formula of the best model is shown below. “*p*” indicates the probability of fox denning at a targeted point.


This formula indicates the probability of denning by red foxes in the Obihiro urban area is high in areas that include low densities of wide roads, narrow roads, and occupied buildings, and a high density of green covered areas within a 500 m radius area.

In the Sapporo study area, higher ranked models produced comparatively stable variables, as shown in Table 3. The lowest AIC value was given for the model with a 300 m radius concentric circle size (Figure 4) and the accuracy of this best model is sufficiently high (AUC = 0.995; Table 4). Extracted variables within the best size of concentric circle were WROAD, OCPBL, RIVER, and GREEN. The directions of effect of these variables were minus for WROAD and OCPBL, and plus for RIVER and GREEN depending on partial regression coefficients (Table 3). The prediction formula of the best model is shown below. “*p*” indicates the probability of fox denning at a targeted point.


This formula indicates that the probability of denning by red foxes in the Sapporo urban area is high in areas that include low densities of wide roads and occupied buildings, and high densities of riverbeds and green covered areas within a 300 m radius area.

**Figure 5:**
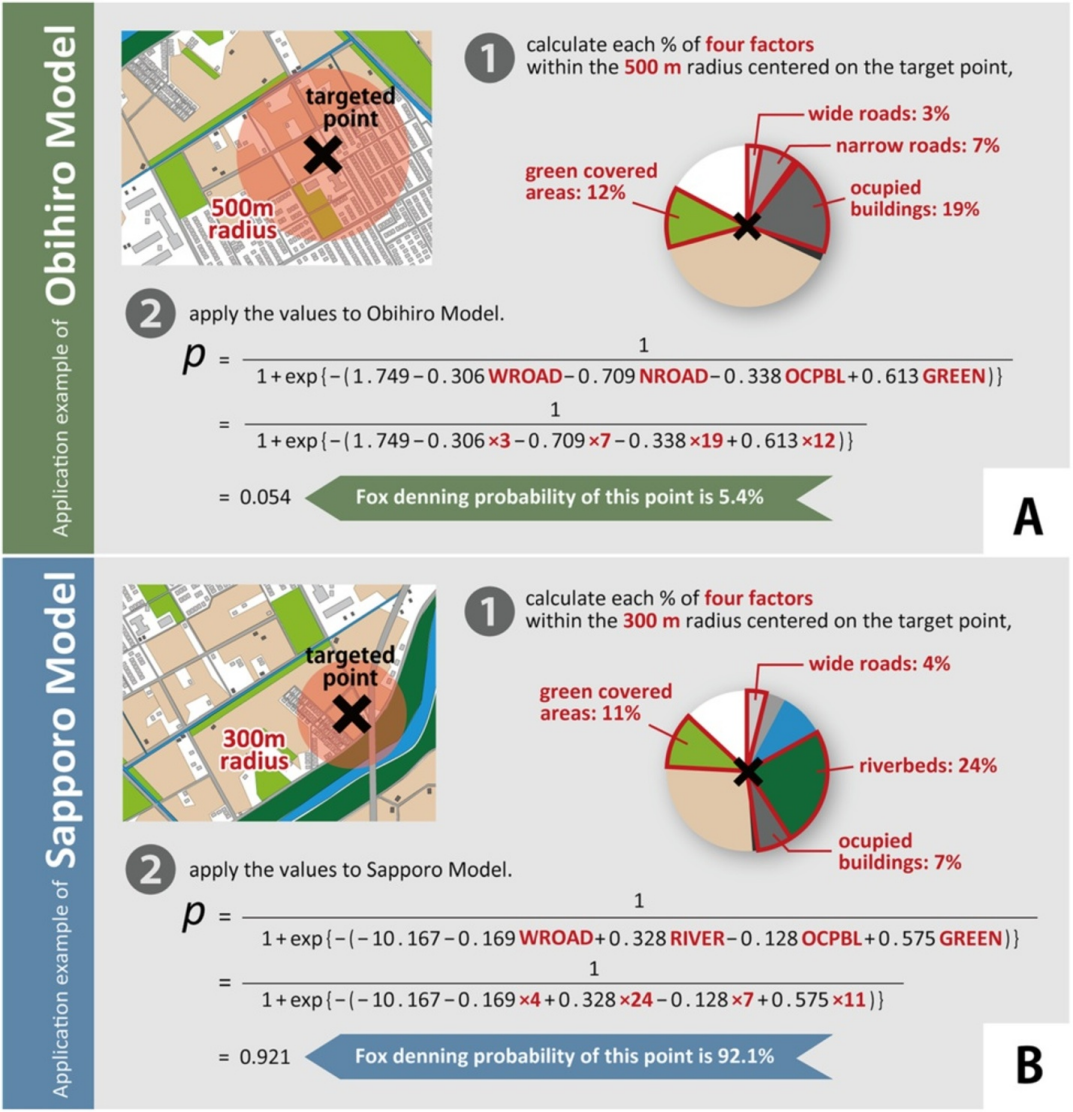
**Application examples of the established models for the two cities.** Panel **A** shows an application example of Obihiro model. If you want to know the probability of fox denning at a targeted point in the urban area of Obihiro City, 1) calculate each percentage of wide roads, narrow roads, occupied buildings, and green covered areas within the 500 m radius centered on the point, 2) apply the values to the model, and then, you can get the answer of 5.4% of probability. Panel **B** shows an application example of Sapporo model. If you want to know the probability of fox denning at a targeted point in the urban area of Sapporo City, 1) calculate each percentage of wide roads, occupied buildings, riverbeds, and green covered areas within the 300 m radius centered on the point, 2) apply the values to the model, and then, you can get the answer of 92.1% of probability.

Application examples of these two models are shown in Figure 5. The confidence intervals of the regression coefficients are shown in Additional file 1 and Additional file 2.

### Traditional analyses

#### Single point analysis

The tendency of den site distribution was examined by analysis using a single point habitat. Out of the 35 dens found in Obihiro, 16 (45.7%) were on “riverbed”, 16 (45.7%) were in “green covered area”, and the remaining 3 (8.6%) were in “farmland” (Table 5). Out of all 65 dens found in Sapporo, 37 (56.9%) were in “green covered area”, 11 (16.9%) were in urban “farmland”, 9 (13.8%) were on “riverbed”, and the remaining 8 (12.3%) were in “vacant building” (Table 6).

**Table 5:** **Distribution pattern of dens and control points per single point habitat in Obihiro study area**

Single point habitat	Number of den sites (%)		Number of control points (%)	
Wide road	0	(0.0)	-	-
Narrow road	0	(0.0)	-	-
Water place	0	(0.0)	-	-
Riverbed	16	(45.7)	13	(10.8)
Occupied building	0	(0.0)	-	-
Vacant building	0	(0.0)	19	(15.8)
Farmland	3	(8.6)	25	(20.8)
Green covered area	16	(45.7)	28	(23.3)
Blank space	0	(0.0)	35	(29.2)
Total	35	(100)	120	(100)

G = 48.947, *p <* 0.0001.

**Table 6:** **Distribution pattern of dens and control points per single point habitat in Sapporo study area**

Single point habitat	Number of den sites (%)		Number of control points (%)	
Wide road	0	(0.0)	-	-
Narrow road	0	(0.0)	-	-
Water place	0	(0.0)	-	-
Riverbed	9	(13.8)	72	(9.9)
Occupied building	0	(0.0)	-	-
Vacant building	8	(12.3)	160	(21.9)
Farmland	11	(16.9)	122	(16.7)
Green covered area	37	(56.9)	175	(24.0)
Blank space	0	(0.0)	201	(27.5)
Total	65	(100)	730	(100)

G = 57.005, *p <* 0.0001.

#### Linear distance analysis

The “key factors” for den site selection were determined from comparison of linear distance variables between den points and control points. Foxes in the Obihiro study area preferred places near “riverbed” (L-river) (*p =* 0.0037) and “green covered area” (L-green) (*p =* 0.0027) as den sites. No significant differences were found in the distance to “wide road” (L-wroad) (*p =* 0.9676), “narrow road” (L-nroad) (*p =* 0.9216), “water place” (L-water) (*p =* 0.0990), “occupied building” (L-ocpbl) (*p =* 0.0719), “vacant building” (L-vctbl) (*p =* 0.9488), “farmland” (L-farm) (*p =* 0.8552), or “blank space” (L-blank) (*p =* 0.0673) from dens (Table 7). Foxes in the Sapporo study area preferred places near “riverbed” (L-river) (*p <* 0.0001), “farmland” (L-farm) (*p <* 0.0001), or “green covered area” (L-green) (*p <* 0.0001) as den sites. No significant differences were found in the distance to “wide road” (L-wroad) (*p =* 0.3091), “narrow road” (L-nroad) (*p =* 0.5728), “water place” (L-water) (*p =* 0.7242), “occupied building” (L-ocpbl) (*p =* 0.3728), “vacant building” (L-vctbl) (*p =* 0.0941), or “blank space” (L-blank) (*p =* 0.3470) from dens (Table 8).

**Table 7:** **Average distances (±SD) from the nearest landscape feature to dens and control points in Obihiro**

Linear distance parameter	Den site		Control point		*** p***
	Average distance (m)	n	Average distance (m)	n	
L-wroad	173 (±132)	35	167 (±103)	120	0.9676
L-nroad	64 (±56)	35	61 (±51)	120	0.9216
L-water	141 (±185)	35	157 (±225)	120	0.0990
L-river	288 (±387)	35	722 (±1003)	120	0.0037*
L-ocpbl	200 (±238)	35	195 (±108)	120	0.0719
L-vctbl	110 (±93)	35	109 (±86)	120	0.9488
L-farm	468 (±390)	35	531 (±480)	120	0.8552
L-green	115 (±176)	35	272 (±309)	120	0.0027*
L-blank	246 (±168)	35	222 (±248)	120	0.0673

*p < 0.05.

*p* indicates the significance probability when the den sites and control points were compared by Mann–Whitney *U* test.

**Table 8:** **Average distances (±SD) from the nearest landscape feature to dens and control points in Sapporo**

Linear distance parameter	Den site		Control point		***p ***
	Average distance (m)	n	Average distance (m)	n	
L-wroad	306 (±364)	65	196 (±121)	730	0.3091
L-nroad	86 (±75)	65	77 (±42)	730	0.5728
L-water	370 (±514)	65	362 (±591)	730	0.7242
L-river	1024 (±996)	65	2446 (±1766)	730	< 0.0001*
L-ocpbl	152 (±130)	65	143 (±84)	730	0.3728
L-vctbl	111 (±108)	65	79 (±57)	730	0.0941
L-farm	352 (±661)	65	1747 (±1364)	730	< 0.0001*
L-green	155 (±425)	65	178 (±197)	730	< 0.0001*
L-blank	106 (±89)	65	93 (±75)	730	0.3470

*p < 0.05.

*p* indicates the significance probability when the den sites and control points were compared by Mann–Whitney *U* test.

## Discussion

In this study, we established a new spatial model to specify the potential habitat of urban red fox dens with a view to preventing contamination by *Echinococcus multilocularis* eggs. This model detects the first priority of environmental requirements for den site selection by red foxes in urban areas to identify the most efficient locations for delivering anthelmintic baits. Our approach focused on the den distribution of red foxes, the definitive host, which differs from previous studies that focused on observed cases of infection in humans or foxes. Spatial modeling was established by modifying a general modeling method commonly used for arthropods. A discussion is presented below for the differences between the general modeling and our new modeling.

In addition to establishment of the new spatial model, we conducted two traditional habitat analyses to compare the tendencies of denning requirements between urban foxes in the present study and non-urban foxes from previous studies.

### Fox den site selection models

#### Interpretation of the models

Obihiro City model suggested that red foxes pay attention to the environment within a 500 m radius from their den sites, and prefer low densities of wide roads, narrow roads, and occupied buildings, and a high density of green covered areas within this range. For Sapporo City, red foxes pay attention to the area within a 300 m radius and prefer low densities of wide roads and occupied buildings, and high densities of rivers and green covered areas.

The difference in size of the heeding range for denning between the two cities may come from differences in sensitivity to the surroundings depending on the degree of urbanization, although this cannot be judged from the present study. Although the size of the ranges differed greatly between the two cities, foxes commonly focused on the densities of wide roads, occupied buildings and green covered areas for their den sites even if the degrees of urbanization were different (Table 2 and 3, Figure 4).

In regard to the preference for sites with a high proportion of green covered areas, this was considered reasonable and appropriate because vegetated ground is easy to dig for denning [38], and the canopy will protect the den from direct sunlight and rain [41, 65]. Furthermore, a vegetated environment will have a high density of prey animals compared with artificial landscapes and thickets prevent easy access by humans. The avoidance of areas with a high density of wide roads and occupied buildings may arise from the low proportion of green covered areas in such areas. Alternatively, a high density of wide roads in their core living area may raise the risk of car accidents. In the present study, the categories “OCPBL” and “VCTBL” were purposely separated in order to clarify the ecological implication of building structures for red foxes, and the result of ignoring vacant buildings suggests that red foxes are nervous about the presence of occupants, not just building structures. In fact, some dens were observed in abandoned barns in Sapporo City. The tolerance to building structures could be developed in the Sapporo population and vacant buildings may provide acceptable environments, which save effort of digging dens and can be even beneficial as a shelter from invaders, such as crows and raptores hunting cubs.

The avoidance of areas with a high density of narrow roads was confirmed only in Obihiro City. For narrow roads, the main users are not cars but pedestrians, bicycles, and dogs accompanied by owners. In the present study areas, foxes tended to avoid people walking or cycling but not cars as potential invaders. The presence of humans and dogs negatively affects denning activity (unpublished data). This result may be affected by varying degrees of tolerance by red foxes toward humans and dogs depending on the degree of urbanization of their territories, which was considered a prevailing reason why the heeding range for denning is larger in Obihiro City than in Sapporo although further research is needed on this topic.

The preference for riverbed areas was confirmed only in Sapporo City. Riverbeds have similar advantages to green covered areas for foxes. The study area in Sapporo City had a much lower proportion of green covered areas than that of Obihiro City, hence, it was suspected that they select riverbed areas to compensate for the lack of the most suitable habitat.

#### Modifications involved for the new modeling approach

The general modeling method required modifications as discussed in order to extract environmental factors for denning requirements for this mid-sized mammal in the micro-habitats of urban landscapes.

### Point 1. Targeting “presence or absence” of dens, not “abundance” of individuals nor dens

Models of potential habitats of red foxes within the urban area need to be based on the “presence or absence” of the dens, not on the “abundance” of individuals. In arthropod modeling, it is recommended that the models need to be based on vector abundance rather than simply vector presence [25]. However, this is not applicable to fox den-based modeling at a city level. The unit for red foxes is a family consisting of approximately 4–7 individuals and an exclusive territory maintained by the family members. Hence, the densities of fox individuals on a grid do not represent the suitability of habitat as is the case for arthropods, because fox territories do not overlap each other, and the density of individuals in a territory varies just depending on the family size. Moreover, the size of each territory is always larger than a standard grid on existing maps especially in the area having low fox densities, and the density of individuals is too low to make a comparison. In contrast, the presence of dens represents the habitat suitability for foxes. Dens are the pivot of their territories, and suitable environments for making dens are fundamental to setting up a territory. We used the presence or absence of dens as the target of modeling. Neither the number nor density of dens makes any sense on this modeling because a fox family always owns and maintains multiple dens in a territory.

### Point 2. Setting predictor variables appropriate for urban red foxes

We set new variables used as predictor variables for the logistic regression analysis in this study, although the general modeling method conveniently uses landscape feature categories of existing thematic maps as predictor variables. Our pre-observation study suggested that disturbance is the critical factor for inhabiting of red foxes in urban landscape; however, few landscape categories in existing thematic maps were sufficient to evaluate these factors. Analysis with inappropriate variables will lead the extraction of exact environmental requirements into failure [31]. Hence, more detailed categories focusing on the degree of disturbance and usage for red foxes are necessary to set appropriate variables to extract sufficient environmental requirements for foxes in urban landscapes. We set nine new landscape features as variables for this purpose, and the analytical base map was newly rendered to fit these new variables.

### Point 3. Modeling with an optimal resolution, “key scale”

The “key scale” was detected from multiple concentric circles (see Methods: Step 4), instead of using arbitrary sized grids (resolutions) on the existing thematic maps. An arbitrary grid size has usually been used as a modeling unit in many previous studies; however, it was reported that modeling based on the proper scale for the target species is necessary to extract precise environmental factors [31, 66], or establishing a model based on an arbitrary grid size may lead to over- or under-estimates of potential as habitats [30, 67]. Meanwhile, any grid or polygon on an existing thematic map is not always adoptable to extract the denning requirements of urban red foxes. Our new method solves the problem of the disagreement between arbitrary grid size and actual requisite scale of the target species by determining the scale for each study area in the process of modeling. Our observations suggested that the size of the heeding range for denning can vary depending on their sensitivity to disturbance, for example, foxes in Sapporo City seem to be nervous about smaller ranges than foxes in Obihiro City. Our model determined the heeding ranges as circles of 500 m radius in Obihiro City (Table 2) and of 300 m in Sapporo City (Table 3), as “key scale”, i.e. modeling unit for each city.

### Traditional analyses

#### Comparison with non-urban areas in previous reports

The results of analyses using two traditional methods in the present study and the results in previous papers showed similar tendencies of den site selection by red foxes; however, our new category of the riverbed environment revealed a more precise reason for the preference.

The result of our single point habitat analysis showed that foxes preferred riverbeds, farmlands, and green covered areas as den sites in both Obihiro and Sapporo City (Tables 5 and 6). Indicating preference to riverbeds and green covered areas was reasonable and agree with previous reports [38, 41, 65], as described above, and farmlands assumed to play a similar role in some cases. The preference of this kind of environment was frequently suggested in other landscapes [36–43].

Linear distance analysis in this study showed common tendencies in the two study areas, in that foxes preferred places near riverbeds and green covered areas as their den sites. On the other hand, they did not exhibit any interest in the distance to wide roads, narrow roads, occupied buildings, vacant buildings, water places, or blank spaces (Tables 7 and 8). The preference for vegetated environments was also reported in other non-urban areas, as mentioned above, and in particular their preference for sites in the vicinity of rivers is known [41, 50]. In this study, preference for rivers was high in both cities regardless of the degree of urbanization, whereas a preference for water places was not confirmed (Tables 7 and 8), unlike in primitive forests and rural landscape [38, 41]. In the present study, the categories “water place” and “riverbed” were purposely separated in order to extract the ecological implications of rivers for red foxes. The preference for riverbeds and the disregarding of water places suggests that they are attracted to rivers as a consequence of the river environment (sloping banks and dry sand that enable them to dig easily, few invaders, and many rodents as food, etc.), not just as a source of water.

Although green covered areas were preferred in both study areas, farmland was preferred only in Sapporo City and disregarded in Obihiro City (Tables 7 and 8). Farmland may compensate for the lack of green covered areas in Sapporo City. The different reaction to farmland between foxes in Obihiro and Sapporo City may be caused by different levels of tolerance to disturbance by human activities. In fact, our direct observation of some dens made in farmland in Sapporo City suggested the red foxes have some level of tolerance to farming disturbance, because they came back and remade dens soon in exactly the same places even when the original dens were completely destroyed by farmers. Although these two categories of landscape, green covered area and farmland, could have huge differences in the degree of disturbance by human activities, it is not possible to judge the sensitivity or tolerance of red foxes to farming disturbance in the present study. Another variable that can express the degree of disturbance in farmland could be set to detect the sensitivity of the red foxes.

#### Unsuitability of traditional methods for extraction of factors in urban landscapes

Specification of key environmental factors for red fox den site selection should be conducted depending on the priorities of foxes among the environment variables tested. However, both the single point and linear distance analyses do not allow for determination of the rank order of each variable, although these methods provide a quick means to obtain an overview of the environmental tendencies. The unsuitability of these methods arises from the lack of suitability of variables in a heterogeneous urban landscape and the properties of the statistical tests (see Methods: “Supplemental analyses by traditional methods”).

Variables used for the single point and linear distance analyses are not appropriate to evaluate complex properties of landscape structure in an urban environment. The single point variables oversimplify the heterogeneous urban landscape with only one representative value for each unit to express the fox’s home range. Linear distance variables are also not appropriate for urban landscapes mainly consisting of artificial structures, such as roads and residences, at a high density. In our study areas, the artificial structures were distributed densely and evenly across the study areas; therefore, all points must be automatically located near to these. This is probably the reason why minimum linear distances from the artificial structures to actual den sites and to the random control points are not significantly different. In fact, the new modeling method extracted roads and occupied buildings as important avoiding factors for urban red foxes, whereas the linear distance analysis could not detect these artifacts variables (see Results: “Fox den site selection models” and “*Linear distance analysis”* and, Discussion: “*Interpretation of the models*” and “*Comparison with non-urban areas in previous reports*”). We tried generating models with the significant variables extracted in the linear distance analysis and found it was invalid (R^2^ = 0.128 for Obihiro, R^2^ = 0.298 for Sapporo; Table 4). The discriminating abilities are also low compared with the best models established by use of the percentages of landscape features as predictor variables (AUC = 0.722 for Obihiro, AUC = 0.881 for Sapporo; Table 4).

Univariate analyses such as G-test and Mann–Whitney *U* test can only detect if the individual variables have significance or not. Because the mere detection of significant variables cannot judge the rank order of significance among them, multivariate analysis conducted in this study is necessary for extracting the most contributing variables by detecting the weights (contribution ratios) of individual variables. For example in non-urban landscapes, the landscape components of roads, houses, areas of vegetation, and rivers were listed as most influential environmental factors [36–43, 50]. However, the comparative ranks of these factors were unclear. Our new modeling method can calculate the contribution ratio and relative rank order of each variable. This modeling approach can be adopted for all landscape types, including urban, suburban, rural, or primitive landscapes.

### Future tasks and perspective

Modeling in this study targets the foxes’ habitat use during breeding season. Expansion of the modeling season to non-breeding seasons will contribute to more efficient control of area contamination with *E. multilocularis* eggs. It is known that foxes change their behavior drastically depending on the season. They depart from their dens in autumn to winter and show different resource requirements from spring to summer, which is the middle of the breeding season. Models for breeding and non-breeding seasons are necessary to identify efficient sites for bating throughout the year.

Accumulation of modeling trials in different cities may reveal variations in denning habits among the cities, and it may let us find some patterns shared among generated models. Recognition of the rules of the patterns, for example, the rule that red foxes change denning behavior depending on the degree of urbanization of their territories (see Discussion: “*Comparison with non-urban areas in previous reports*”), may allow us to quickly perform a mission of anthelmintic baiting with a better degree of precision without the laborious modeling process.

Disease control tools must be universal and ubiquitous so that any person under any conditions can use and arrange them as the situation demands and at a low cost. However, a lack of thematic maps for the analysis of mid-sized generalist mammals in urban areas is the biggest problem at present. Quick modeling can be achieved if thematic maps including all variables as we proposed in this study were available. In this study, we used the free software and open-source analysis tools as much as possible, in order to minimize costs. Vector- or transmitter-based modeling can apply to the control of multiple zoonotic diseases from the same vectors or transmitters [25]. Preparing a set of adequate thematic maps for the vectors and transmitters in urban areas is reasonable from this viewpoint as well.

## Conclusions

### Suggestions for anthelmintic baiting strategies for urban red foxes

Anthelmintic baiting needs to be conducted continuously to keep the local fox populations free from parasites. We suggest the effective strategy for it, as listed below.

1. Aim to make the target area be occupied by an uninfected fox population.
2. Deliver anthelmintic bait to the sites with a high probability of fox den presence based on the model by our protocol dedicated to urban fox ecology.
3. Establish the model for every city to adapt the variation of fox denning requirements and accumulate the model patterns.

### Suggestions for the spatial modeling protocol of urban red fox ecology

Establishment of the model for red foxes inhabiting urban landscape requires some unique approaches, as listed below (see also Discussion: *“Modifications involved for the new modeling approach”*: Point 1–3).

1. Targeting “presence or absence” of dens, not “abundance” of individuals nor dens, especially in the area having low fox densities.
2. Setting predictor variables focusing on the degree of disturbance not only usability for red foxes.
3. Detecting the key spatial scale for denning to clarify the appropriate modeling unit instead of applying arbitrary grid size (resolution).

## Electronic supplementary material

Additional file 1: **Confidence intervals of the coefficients of selected variables included in the models in each scale for Obihiro.** (PDF 48 KB)

Additional file 2: **Confidence intervals of the coefficients of selected variables included in the models in each scale for Sapporo.** (PDF 47 KB)
