# Supplementary material for: Where to deliver baits for deworming urban red foxes for Echinococcus multilocularis control: new protocol for micro-habitat modeling of fox denning requirements
Source: Parasit Vectors. 2014 Aug 6;7:357. doi: 10.1186/1756-3305-7-357 (PMC4262088; doi:10.1186/1756-3305-7-357)
Supplement: Supplementary file 1 — Additional file 1: Confidence intervals of the coefficients of selected variables included in the models in each scale for Obihiro. (PDF 48 KB) [file 13071_2014_1631_MOESM1_ESM.pdf]

Additional file 1. Confidence intervals of the coefficients of selected variables included in the models in each scale for Obihiro.

| Variable | 100m | | | 200m | | | 300m | | | 400m | | | 500m | | |
|---|---|---|---|---|---|---|---|---|---|---|---|---|---|---|---|
| | Coefficient | 95%CI | | Coefficient | 95%CI | | Coefficient | 95%CI | | Coefficient | 95%CI | | Coefficient | 95%CI | |
| | | 2.5% | 97.5% | | 2.5% | 97.5% | | 2.5% | 97.5% | | 2.5% | 97.5% | | 2.5% | 97.5% |
| Intercept | 3.456 | 0.583 | 6.861 | 5.616 | 3.213 | 8.776 | 9.205 | 3.864 | 15.670 | -0.005 | -2.813 | 2.700 | 1.749 | -1.266 | 5.057 |
| WROAD | -0.245 | -0.436 | -0.103 | -0.226 | -0.409 | -0.079 | -0.133 | -0.273 | -0.028 | -0.180 | -0.345 | -0.059 | -0.306 | -0.571 | -0.123 |
| NROAD | -0.290 | -0.529 | -0.113 | -0.616 | -0.969 | -0.356 | -0.597 | -1.123 | -0.261 | -0.489 | -0.959 | -0.180 | -0.709 | -1.282 | -0.323 |
| WATER | - | - | - | - | - | - | - | - | - | - | - | - | - | - | - |
| RIVER | - | - | - | - | - | - | - | - | - | - | - | - | - | - | - |
| OCPBL | -0.222 | -0.391 | -0.101 | -0.280 | -0.518 | -0.075 | -0.407 | -0.896 | -0.023 | -0.271 | -0.491 | -0.123 | -0.338 | -0.626 | -0.146 |
| VCTBL | -0.131 | -0.248 | -0.331 | -0.190 | -0.321 | -0.083 | -0.184 | -0.339 | -0.064 | - | - | - | - | - | - |
| FARM | - | - | - | - | - | - | - | - | - | - | - | - | - | - | - |
| GREEN | 0.766 | 0.432 | 1.250 | 0.047 | -0.003 | 0.111 | 0.216 | 0.063 | 0.468 | 0.522 | 0.239 | 0.917 | 0.613 | 0.257 | 1.112 |
| BLANK | -0.162 | -0.317 | -0.032 | - | - | - | - | - | - | - | - | - | - | - | - |

| Variable | 600m | | | 700m | | | 800m | | | 900m | | | 1000m | | |
|---|---|---|---|---|---|---|---|---|---|---|---|---|---|---|---|
| | Coefficient | 95%CI | | Coefficient | 95%CI | | Coefficient | 95%CI | | Coefficient | 95%CI | | Coefficient | 95%CI | |
| | | 2.5% | 97.5% | | 2.5% | 97.5% | | 2.5% | 97.5% | | 2.5% | 97.5% | | 2.5% | 97.5% |
| Intercept | 1.808 | -0.976 | 4.584 | 3.466 | 1.711 | 5.617 | 1.501 | 0.005 | 3.113 | 2.738 | 1.238 | 4.421 | -0.222 | -1.923 | 1.486 |
| WROAD | -0.186 | -0.341 | -0.079 | -0.192 | -0.348 | -0.061 | -0.118 | -0.211 | -0.039 | -0.196 | -0.302 | -0.109 | -0.160 | -0.257 | -0.077 |
| NROAD | -0.514 | -0.931 | -0.230 | -0.563 | -0.885 | -0.322 | -0.302 | -0.503 | -0.152 | -0.196 | -0.323 | -0.093 | -0.132 | -0.265 | -0.020 |
| WATER | - | - | - | - | - | - | - | - | - | - | - | - | - | - | - |
| RIVER | - | - | - | - | - | - | - | - | - | - | - | - | 0.438 | 0.209 | 0.719 |
| OCPBL | -0.201 | -0.348 | -0.088 | -0.159 | -0.283 | -0.052 | -0.147 | -0.252 | -0.054 | -0.049 | -0.115 | 0.015 | -0.054 | -0.106 | -0.012 |
| VCTBL | - | - | - | - | - | - | - | - | - | - | - | - | - | - | - |
| FARM | - | - | - | - | - | - | - | - | - | - | - | - | - | - | - |
| GREEN | 0.275 | 0.115 | 0.534 | 0.041 | -0.002 | 0.094 | 0.145 | 0.054 | 0.263 | 0.141 | -0.041 | 0.346 | 0.203 | 0.058 | 0.363 |
| BLANK | - | - | - | - | - | - | - | - | - | -0.059 | -0.112 | -0.011 | -0.067 | -0.126 | -0.015 |
