# Supplementary material for: Where to deliver baits for deworming urban red foxes for Echinococcus multilocularis control: new protocol for micro-habitat modeling of fox denning requirements
Source: Parasit Vectors. 2014 Aug 6;7:357. doi: 10.1186/1756-3305-7-357 (PMC4262088; doi:10.1186/1756-3305-7-357)
Supplement: Supplementary file 2 — Additional file 2: Confidence intervals of the coefficients of selected variables included in the models in each scale for Sapporo. (PDF 47 KB) [file 13071_2014_1631_MOESM2_ESM.pdf]

Additional file 2. Confidence intervals of the coefficients of selected variables included in the models in each scale for Sapporo.

Variable	100m			200m			300m			400m			500m		
	Coefficient	95%CI		Coefficient	95%CI		Coefficient	95%CI		Coefficient	95%CI		Coefficient	95%CI	
		2.5%	97.5%		2.5%	97.5%		2.5%	97.5%		2.5%	97.5%		2.5%	97.5%
Intercept	-2.265	-4.295	-0.528	-9.526	-14.576	-6.195	-10.167	-16.125	-6.317	-5.822	-10.200	-2.870	-4.343	-7.322	-1.954
WROAD	-0.288	-0.434	-0.178	-0.186	-0.361	-0.063	-0.169	-0.386	-0.031	-0.266	-0.483	-0.124	-0.314	-0.513	-0.170
NROAD	-0.724	-1.132	-0.376	-	-	-	-	-	-	-	-	-	-0.602	-1.030	-0.245
WATER	-	-	-	-	-	-	-	-	-	-	-	-	-	-	-
RIVER	0.141	0.083	0.207	0.344	0.206	0.564	0.328	0.175	0.573	0.192	0.113	0.299	0.115	0.061	0.178
OCPBL	-0.247	-0.391	-0.134	-0.134	-0.287	-0.022	-0.128	-0.331	0.022	-0.273	-0.487	-0.120	-0.093	-0.218	0.008
VCTBL	-	-	-	-	-	-	-	-	-	-1.022	-1.769	-0.472	-	-	-
FARM	-	-	-	-	-	-	-	-	-	-	-	-	-	-	-
GREEN	0.393	0.257	0.583	0.562	0.348	0.919	0.575	0.361	0.907	0.590	0.366	0.959	0.476	0.320	0.695
BLANK	-	-	-	-	-	-	-	-	-	-	-	-	-0.061	-0.129	0.000

Variable	600m			700m			800m			900m			1000m		
	Coefficient	95%CI		Coefficient	95%CI		Coefficient	95%CI		Coefficient	95%CI		Coefficient	95%CI	
		2.5%	97.5%		2.5%	97.5%		2.5%	97.5%		2.5%	97.5%		2.5%	97.5%
Intercept	-8.018	-11.124	-5.725	-2.783	-4.959	-0.945	-4.326	-6.385	-2.680	-8.850	-11.851	-6.535	-2.409	-4.498	-0.595
WROAD	-0.474	-0.947	-0.084	-0.330	-0.547	-0.167	-0.263	-0.451	-0.138	-0.047	-0.099	0.001	-0.268	-0.424	-0.146
NROAD	-	-	-	-	-	-	-0.353	-0.725	-0.030	-	-	-	-0.846	-1.220	-0.522
WATER	-	-	-	-	-	-	-	-	-	-	-	-	-	-	-
RIVER	0.338	0.227	0.486	0.144	0.089	0.211	0.147	0.089	0.216	0.147	0.096	0.207	0.127	0.075	0.184
OCPBL	-0.235	-0.393	-0.121	-0.300	-0.481	-0.156	-0.238	-0.394	-0.117	-0.259	-0.450	-0.090	-0.270	-0.420	-0.148
VCTBL	-	-	-	-1.016	-1.515	-0.619	-	-	-	-	-	-	-	-	-
FARM	-	-	-	-	-	-	-	-	-	-	-	-	-	-	-
GREEN	0.422	0.292	0.601	0.413	0.287	0.589	0.380	0.265	0.545	0.645	0.470	0.878	0.419	0.285	0.603
BLANK	-	-	-	-	-	-	-	-	-	-0.407	-0.774	-0.080	-	-	-
